# The pedagogical value of near-peer feedback in online OSCEs

**DOI:** 10.1186/s12909-022-03629-8

**Published:** 2022-07-25

**Authors:** Julia Sader, Bernard Cerutti, Louise Meynard, Frédéric Geoffroy, Véronique Meister, Adeline Paignon, Noëlle Junod Perron

**Affiliations:** 1 Unit of Development and Research in Medical Education, Faculty of Medicine, University of Geneva, Rue Michel-Servet 1- CMU 5-6, Geneva, Switzerland; 2 Interprofessional Centre of Simulation - CIS, Geneva, Switzerland; 3 HES-SO University of Applied Sciences and Arts of Western Switzerland, School of Health Sciences Geneva, Geneva, Switzerland

**Keywords:** Near-peer, Feedback, Online, OSCE

## Abstract

**Purpose of the article:**

During the Covid-19 pandemic, formative OSCE were transformed into online OSCE, and senior students (near peers) substituted experienced clinical teachers. The aims of the study were to evaluate quality of the feedbacks given by near peers during online OSCEs and explore the experience of near-peer feedback from both learner’s and near peer’s perspectives.

**Materials and methods:**

All 2nd year medical students (*n* = 158) attended an online OSCE under the supervision of twelve senior medical students. Outcome measures were 1) students’ perception of the quality of the feedback through an online survey (Likert 1–5); 2) objective assessment of the quality of the feedback focusing on both the process and the content using a feedback scale (Likert 1–5); 3) experience of near peer feedback in two different focus groups.

**Results:**

One hundred six medical students answered the questionnaire and had their feedback session videotaped. The mean perceived overall quality of senior students’ overall feedback was 4.75 SD 0.52. They especially valued self-evaluation (mean 4.80 SD 0.67), balanced feedback (mean 4.93 SD 0.29) and provision of simulated patient’s feedback (mean 4.97 SD 0.17). The overall objective assessment of the feedback quality was 3.73 SD 0.38: highly scored skills were subjectivity (mean 3.95 SD 1.12) and taking into account student’s self-evaluation (mean 3.71 (SD 0.87). Senior students mainly addressed history taking issues (mean items 3.53 SD 2.37) and communication skills (mean items 4.89 SD 2.43) during feedback. Participants reported that near peer feedback was less stressful and more tailored to learning needs– challenges for senior students included to remain objective and to provide negative feedback.

**Conclusion:**

Increased involvement of near peers in teaching activities is strongly supported for formative OSCE and should be implemented in parallel even if experience teachers are again involved in such teaching activities. However, it requires training not only on feedback skills but also on the specific content of the formative OSCE.

**Supplementary Information:**

The online version contains supplementary material available at 10.1186/s12909-022-03629-8.

## Table A – practice points

**Table:** 

Practice points	• Near peers, with limited training in teaching skills, can be considered as valuable and credible sources of feedback • Near peer feedback is experienced as less stressful and more tailored to students’ needs • It represents a learning opportunity for near peers • Teaching of more complex skills still requires the presence of experienced tutors

## Introduction

Feedback is an essential component of medical education. Formative feedback is defined as an information given to the learner with the intention of adjusting his or her thinking or behavior for the purpose of improving learning [1]. It is the most widely used approach to stimulate learning and development at all levels of clinical expertise development [2]. It is used in formative objective structured clinical examinations (OSCEs) to help medical students improve their clinical skills such as history taking, physical examination and communication skills [3]. It is also widely used in the workplace, with the global shift towards competency-based curricula and programmatic assessment during both pre-graduate, graduate and continuous training [4–6].

In order to be effective, feedback should be specific, timely, and credible. It should be based on observable behavior and in response to a problem or a task, and promote a specific and actionable goal [1, 7, 8]. Effective feedback is not about just delivering a message; it is described a conversation in which both the supervisor and the student collaboratively reflect on his/her performance and how to improve it [9, 10]. Feedback effectiveness also depends on students’ individual receptiveness which is in turn influenced by their motivations, fears, expectations as well as the credibility of the feedback provider [11]. They will all impact on students’ acceptance and interpretation of feedback [12, 13]. Credibility is a broad construct and refers to dimensions such as trustworthiness, accuracy, believability, reliability, intention of the feedback provider but also to features such as age, gender, experience, expertise and professional background [13]. Some studies evaluated the quality of feedback according to the clinical teachers’ features (gender, seniority, and specialty) [14–18].

Do peer or near peer students are credible as feedback providers and provide high quality feedback? A near-peer tutor is “a trainee one or more years senior to another trainee” while a peer-tutor is one at the same level [19]. Peer and near-peer teaching (NPT) has become an increasingly recognized method for teaching and learning within medical education [20]. It is aligned with social constructivism which promotes learning in a social setting where individuals help each other through a shared culture of knowledge [21]. It is also fits cognitive congruence theory as near-peer teachers usually better understand learner needs since the gap in knowledge between a senior and a junior student is smaller than between an experienced tutor and a student [22, 23].In peer-assisted learning in medical education, the most common topics are the physical examination skills and OSCE [20]. A recent scoping review about peer assessment in OSCE revealed that peer examiners provided valuable feedback [24]. However, in most studies, feedback quality was assessed through students’ perceptions using questionnaires or Likert scales but was not objectively assessed.

The Covid-19 pandemic had two major impacts on OSCEs. First, in several settings, face to face OSCE were transformed into online OSCEs [25, 26]. Peer and near peer involvement in teaching increased and gained visibility [27, 28]. In our setting, the in-person formative OSCEs were transformed into online OSCEs, and senior medical students replaced the experienced clinical teachers who were no longer were available to supervise formative OSCEs given the amount of clinical work at the hospital.

The aims of the study were 1) to evaluate the perceived quality of feedback given by near peers during an online OSCEs 2) to objectively assess the quality of near peer feedback and compare it with the quality of feedback given by experienced clinical teachers during an face to face OSCE a few years earlier; 3) to explore medical students junior (year (Y) 2 learners) and senior (Y4-5 tutors) experiences of receiving from and giving feedback as near peers.

## Material and methods

### Design and setting

A prospective mixed method study was conducted to investigate the quality and added value of near peer feedback at the Faculty of Medicine, Geneva University, Switzerland. The Geneva Faculty of Medicine offers a 6-year curriculum divided into 3 pre-clinical years (bachelor) and 3 clinical years (master) to 158 medical students (the total n of our students in the medical school). Clinical skills training occurs during the 2^nd^ and 3^rd^ bachelor years. During these two years, medical students have the opportunity to practice history taking, physical examination and communication skills during four formative OSCEs focusing successively on different topics (abdominal, cardiac, respiratory, neurological) which are usually organized in three formats: 1) *a direct observation format* – direct observation of the student – standardized patient interaction followed by an oral feedback given by a clinical teacher 2) *a video based format* – a delayed oral feedback given by a clinical teacher based on the observation of the videotaped student- standardized patient interaction; 3) *a group format*—direct observation followed by an oral feedback involving 1 clinical teacher in a general practice setting and 3 students –three students interact consecutively with a standardized patient mimicking a different clinical problem, followed by a group (clinical teacher, peer and simulated patient) feedback.

Clinical teachers are generally 20–30 experienced physicians who have both clinical and teaching activities.

As the Covid-19 pandemic outbreak occurred, the medical school closed its doors mid of March 2020: face to face seminars were cancelled, clinical teachers, mostly working in hospital settings became unavailable and the clinical skills training team had to adapt the formative OSCE to such constraints.

#### Participants

All 2^nd^ year medical students were invited to attend the new online version of the 2^nd^ formative OSCE (*n* = 158). Twelve senior medical students (4^th^ and 5^th^ year) were asked to replace the clinical teachers. They were part of near peer tutors already involved in the teaching of physical examination during years 2 and 3 (17 seminars).

#### Procedure

The formative OSCE station focused on a cardiac topic. All medical students received a link to attend an online formative OSCE (via zoom, a videoconference platform providing face views) [29] during which students by group of two successively interacted with the patient mimicking two different clinical problems (stable angina, heart failure). During the 20 min, they were asked to collect information, describe loud the different steps of the physical examination, briefly explain their clinical hypothesis and end the encounter. The encounter was followed by a 20-min group feedback including senior medical student, peer and simulated patient feedback before the next student started interacting with the patient.

Senior medical students received a one-hour interactive training on how to give feedback and a one-hour training on the learning objectives of the OSCE and how to use the online platform prior to the online OSCE. They received a checklist form to assess the different items expected for history taking, physical examination and communication skills.

All feedback sessions were videotaped. After the session, medical students received an online questionnaire including an information and consent sheet to be signed.

#### Outcomes measures

- 1. Online questionnaire to students on perceived quality of feedback
- Online questionnaire on the perceived quality of the feedback – after the formative OSCE; students received a 15-item online questionnaire (Likert scale 1-5) evaluating the perceived quality of the feedback received. The questions addressed the usefulness of the feedback for improving clinical skills (history taking, physical examination and communication) as well as on different elements of the feedback process. The items derived from a grid used from a previous study that confirmed its ability to discriminate between poor and good feedback givers [16]. The content of the grid was developed on the basis of a literature review on feedback principles and strategies [16, 30–33].
- 2. Objective assessment of the quality of feedback (analysis of videotaped feedback)
- The feedback quality – the quality of the feedback given exclusively by the senior student was objectively assessed through the analysis of the videotaped feedback sessions using a feedback scale focusing both on the content and process of feedback. It included seven content items about history taking, physical examination and communication elements as well as elaboration on clinical reasoning and communication/professionalism issues. Elaboration referred to whether the senior student addressed in facilitative or directive way the importance or relevance of collecting some items during the feedback session (e.g. “Why it is important to ask about thromboembolic risk factors in a woman complaining with chest pain?” or “Do not forget to explore patients’ beliefs and emotions: it will influence the way you will explain the diagnosis!”). The 14 feedback process items derived from a validated feedback scale used in previous studies [16, 30] that follows the structure of the MAAS-Global, a well-known communication skill coding instrument, given the close similarities existing between a clinical encounter and a teaching encounter [30, 34]. These instruments included specific elements of the feedback process, 3 transversal dimensions (empathy, pedagogical effectiveness, structure) as well there is 1 for the global rating (Table 1). In order to analyze the quality of videotaped feedback, we used a coding book that provides, for each feedback item, the precise definition and examples of the five anchors of the Likert scale (1 to 5). This coding book is available upon request. NJP, VM and LM first independently coded the first 12 feedback sessions using the coding book and discussed their coding in order to ensure a correct understanding of the coding definitions. Then, LM coded the remaining videotaped feedback sessions. Interrater reliability of coding, measured by blind coding (NJP) of 10% of the videotaped sessions, was good (intraclass correlation coefficient =0.88).

**Table 1:** Objective analysis of the feedback quality in 2020 (online feedback given by near peer students) compared to 2013 (face to face feedback given by experienced clinical teachers)

Objective analysis of the feedback quality	Senior students Online feedback	Experienced tutors Face to face feedback	
	2020 *N* = 106	2013 *N* = 37	
	Mean (SD) Likert scale 1–5	Mean (SD) Likert scale 1–5	*p* value^a^
The tutor explored students’ learning needs	3.42 (0.76)	2.14 (1.73)	< 0.0001
The tutor stimulated students’ self-assessment	3.27 (0.97)	1.73 (1.50)	< 0.0001
The feedback was descriptive	3.41 (0.69)	3.68 (1.00)	0.096
The feedback was subjective	3.95 (1.12)	2.49 (2.00)	0.0001
The feedback was balanced (between both the positive and constructive feedback)	3.34 (1.02)	3.57 (1.26)	0.328
The supervisor took into account the student’ s self-assessment	3.71 (0.87)	2.00 (1.99)	< 0.0001
The tutor stimulated students to participate to the problem-solving process	3.07 (0.42)	2.70 (1.41)	0.007
The tutor used role playing or hands on	1.39 (0.84)	0.95 (1.49)	< 0.0001
The tutor checked students’ understanding	3.39 (1.13)	2.09 (1.72)	< 0.0001
Transversal dimensions			
Empathy	5.59 (0.51)	3.81 (1.05)	< 0.0001
Pedagogical effectiveness	3.81 (0.69)	2.78 (1.57)	0.0002
Structure of the feed-back	3.63 (0.48)	2.49 (1.33)	< 0.0001
Verbal interaction	3.15 (0.36)	3.27 (0.96)	0.6629
Global evaluation (sum of the scores of items)	3.73 (0.38)	2.93 (1.23)	0.0002

^a^Wilcoxon rank sum test
- Both the questionnaire on the perceived quality of the feedback and the feedback scale had been used in a previous study in 2013 that included 2nd and 3rd year medical students and clinical teachers [16]. It was used to evaluate whether the content and process of feedback varied according to the tutors’ profile (generalist versus specialist clinical teachers).
- 3. Focus groups about students and senior students’ experiences of near peer feedback
- We conducted 2 focus groups (one with Y2 students and one with Y4-5 tutors) via the same videoconference platform with a convenient sample of students to deepen our understanding of the perception of online OSCEs and near-peer teaching feedback. Focus groups are a group discussion which is moderated by a researcher, such groups are used for generating information regarding the participant’s experiences and beliefs about a particular topic [35]. The focus groups guide included several questions about participants’ perceptions as near-peer feedback receivers and givers and their experience of the online formative station (see Appendix A). External moderators (JS and LM), who were not involved in the organization and implementation of the online OSCE and had no professional relationship with the participants led the discussion in order to make participants feel free to express their views without any hierarchical pressure. The sessions were audiotaped and transcribed ad verbatim. We selected the answers from four question for the purpose of the study.

#### Analysis

Perceived feedback quality data as well as objective feedback process (items measured using Likert’s scale) and content (occurrence of comments) data were summarized by means and standard deviations. We compared students’ perception of the quality of feedback as well as the objective analysis of feedback content and process between 2020 (an online OSCE supervised by senior students) (Table 2) and 2013 (face to face OSCE supervised by experienced tutors), as we had conducted a study assessing the quality of feedback during this year using the same questionnaires and feedback scales [16, 30]. Both 2013 and 2020 OSCE focused on cardiac symptoms. However, the physical examination approach (performance in 2013 vs description in 2020) and the format (face to face vs online) were different. Potential content differences were investigated using Wilcoxon ranks sum tests. All analyses were run on R 3.5.2 (the R Foundation for Statistical Computing, Vienna, Austria).

**Table 2:** Feedback content in 2020 (online feedback given by near peer students) compared to 2013 (face to face feedback given by experienced clinical teachers)

	2020 *N* = 106	2013 *N* = 37	*p* value^a^
Global performance	0.30 (0.46)	0.84 (0.90)	0.0002
Content—History taking	3.53 (2.37)	5.11 (3.16)	0.0068
Content—Physical examination	1.71 (1.62)	4.38 (2.72)	< 0.0001
Content—Explanation-end	0.84 (0.95)	0.92 (0.92)	0.5584
Process—Communication skills	4.89 (2.43)	4.70 (3.51)	0.3376
Elaboration^b^- clinical reasoning	0.78 (1.09)	0.70 (0.91)	0.9534
Elaboration^b^- communication/professionalism	0.70 (0.86)	1.32 (1.42)	0.0286

^a^Wilcoxon rank sum test

^**b**^Elaboration: number of times the near peer/clinical teacher elaborated in directive or facilitative way on the importance or relevance of collecting such items during the feedback session

For the focus groups, a thematic analysis was conducted to explore the different themes which emerged from the data [36, 37]. The transcripts were first read by all authors, who then met to discuss their observations and develop a list of codes. Codes were developed to reflect the discussion questions and focused on participants’ perceptions and experiences of near-peer feedback during online OSCEs. Then, JS coded all transcripts using ATLAS Ti [38] and only the quotes in relation with near-peer feedback were selected and then translated into English by a native English speaker (JS). The intercoder reliability was checked by having an interactive cross-check coding amongst all authors. To maintain the highest inter-coder reliability the two main researcher (NJP and JS) coded separately. An agreement was achieved for > 80% of the coding and disagreement was solved through discussion.

## Results

A hundred and six 2^rd^ year medical students filled in the questionnaire and had their feedback session videotaped (participation’s rate = 67%). Eleven senior medical students supervised the formative OSCE and gave feedback.

### Quality of near peer feedback

Students felt that the formative OSCE helped them improve their clinical skills (mean score > 4) except for physical examination skills that could only be assessed through description (Table 3). Students’ perception of the feedback was very good with all scores above 4 except for opportunities to practice parts of the encounter during feedback (Table 3). They especially appreciated the fact that senior students were aware of their learning needs, made them feel comfortable, gave balanced feedback, involved them actively in the self-evaluation and problem-solving phases and involved the simulated patient in the feedback. Their evaluation of quality of feedback were statistically significantly higher than students’ ratings documented in 2013 when feedback was given by experienced clinical teachers during face-to-face OSCE.

**Fig. 1:**
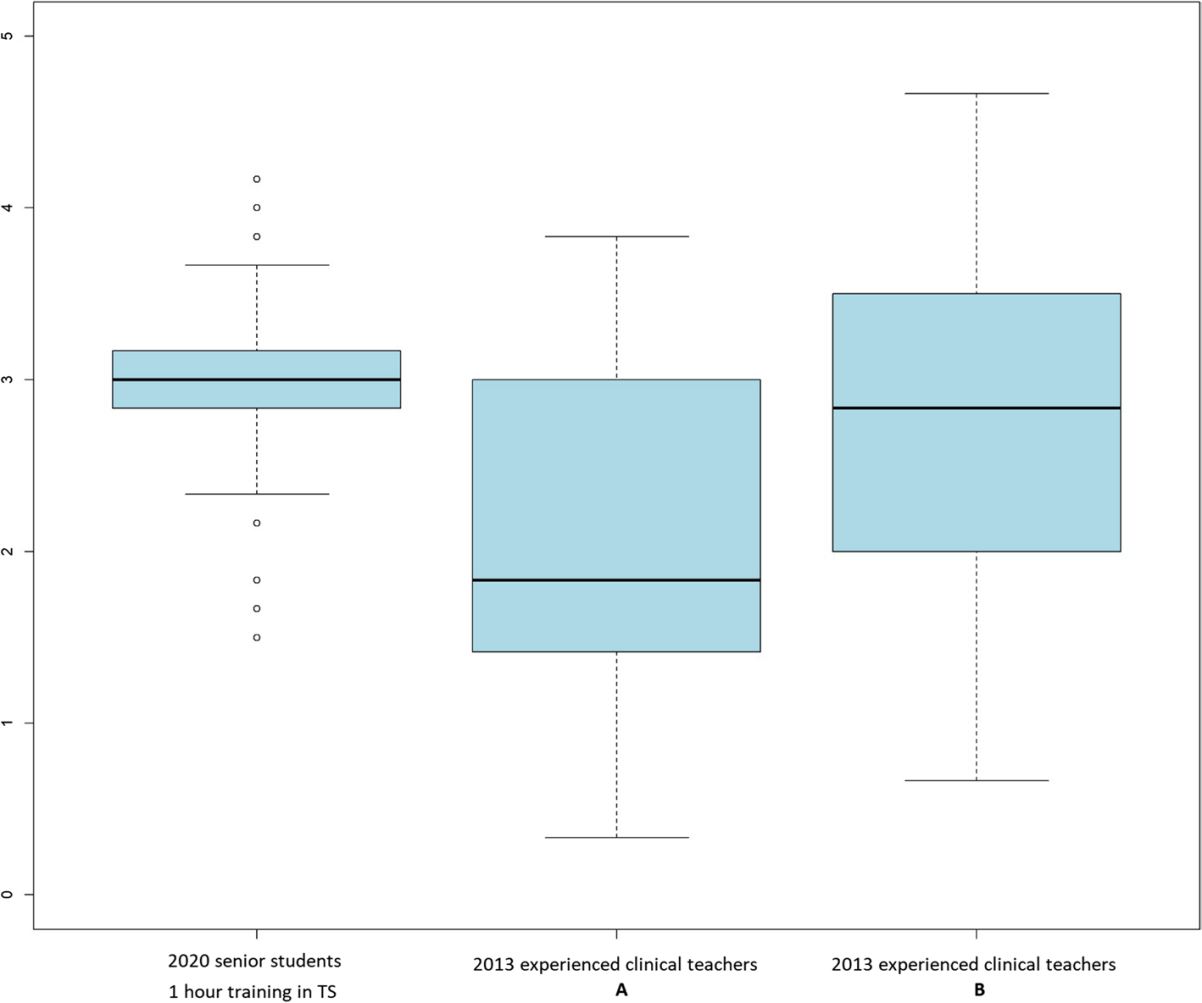
Global feedback score assessed objectively (Likert scale 1–5, 1 = poor and 5 = excellent) of 2020 senior students and 2013 clinical teachers (A with no prior training in teaching skills – B with prior training in teaching skills)

Regarding the feedback process, objective analysis showed that senior students actively involved students in feedback, (students’ exploration of learning needs, self-assessment, active participation in problem-solving, checking for understanding) and rarely provided opportunities to practice parts of the history or communication skills during the feedback session (Table 1). They performed statistically significantly much better than experienced clinical teachers (in 2013) in all phases of the feedback process except regarding feedback balance (Table 1). Figure 1 showcases the overall global feedback scores of senior students (2020) and of experienced clinical teachers (2013) further divided into two sub groups: experienced clinical teachers with no prior training in teaching skills (group A) and with prior training in teaching skills (group B), The senior students’ quality of their feedback was of the same level as and had more homogeneity than the one delivered by experienced clinical teachers trained in teaching skills (group A) in face-to-face OSCE 7 years ago (Fig. 1).

**Table 3:** Students’ perceptions of the feedback quality in 2020 (online feedback given by near peer students compared to 2013 (face to face feedback given by experienced clinical teachers)

Students’ perceptions of the feedback	Senior students Online feedback	Experienced tutors Face to face feedback	
	2020 *N* = 107	2013 *N* = 64	
	Mean (SD) Likert 1–5	Mean (SD) Likert 1–5	*p* value^a^
The feedback session was useful	4.75 (0.52)	4.35 (0.93)	0.0022
I improved my history taking skills	4.64 (0.57)	4.07 (1.03)	< 0.001
It improved my physical examination skills	3.36 (1.24)	4.11 (0.99)	0.00012
I improved my communication skills	4.56 (0.73)	4.00 (1.21)	0.00014
The tutor was aware of what I needed to learn	4.83 (0.42)	4.46 (0.86)	0.0006
The tutor made me feel comfortable and confident	4.93 (0.26)	4.25 (1.15)	< 0.0001
The tutor asked me my learning needs	4.23 (1.31)	2.69 (1.82)	< 0.0001
The tutor asked me to evaluate what I did well	4.79 (0.67)	3.91 (1.27)	< 0.0001
The tutor asked me to evaluate what I could improve	4.80 (0.67)	4.31 (1.00)	< 0.0001
The tutor gave me balanced feedback (including both positive and less positive aspects)	4.93 (0.29)	4.38 (0.90)	< 0.0001
The tutor stimulated me to participate to the problem-solving process	4.36 (0.92)	3.17 (1.50)	< 0.0001
The tutor gave me precise and concrete suggestions for improvement	4.71 (0.63)	4.03 (1.25)	< 0.0001
The tutor provided me opportunities to practice parts of the history taking, physical exam or the communication	3.50 (1.46)	2.00 (1.53)	< 0.0001
The tutor asked the simulated patient to give me feedback	4.97 (0.17)	3.92 (1.67)	< 0.0001
The tutor checked my understanding	3.84 (1.37)	2.38 (1.54)	< 0.0001

^a^Wilcoxon rank sum test

In terms of content, senior students addressed less elements in relation to history taking and physical examination and expressed less global comments about performance. Their teaching focused less on elaboration of communication/professionalism dimensions but addressed clinical reasoning in the same amount than experienced tutors/supervisors.

The mean duration of direct observation-based feedback (isolated from peer and standardized patient feedback) was however longer for senior students (8.90 min (SD 4.6)) than for experienced clinical teachers (6.8 min (SD 3.4)).

### Students and senior students’ experiences of near-peer feedback

Out of 158 2^nd^ year students, 5 were included in the student focus groups. Out of the 12 senior students, 8 took part (5 from 4th and 3 from 5th year). Reasons for non-response were not recorded.

#### Less stressful and more tailored to students’ needs

Students reported that learning from peers was experienced as less threating and more tailored to their needs because senior students were more aware of their learning needs and the stress, they could experience during formative OSCEs.

*“On the contrary, I thought it was good. Because as she is also a student, she also had some tips to give us, techniques if we made a mistake.” (Student 1)*

“*Because the students they also know more of how it’s going to be on the exam. More tailored to our needs. I also like to have feedback from a doctor.” (Student 4)*

However, they perceived near peer feedback to be complementary to clinical teachers’ feedback.

*I like the doctor because he has experience and he talks more about anecdotes or real things. You can see that he knows the subject really well. And then I like the students. Because often it’s more precise, they prepare better for the OSCE. (Student 2)*

Senior student tutors expressed similar thoughts and considered that they could guide more explicitly the student in the learning process and make the session less stressful and more interactive.

*“And I think it can be quite reassuring to be in front of students, for a first experience.”(Senior student 8)*

*“It was more interactive. They weren’t afraid to ask more questions.” (Senior student 5)*

#### Different focus

Some senior students reported being less focused on specific elements of the history taking or physical examination parts, making their feedback therefore less clinic oriented. In the senior students’ previous experience as juniors, they felt more emphasis was needed on the process of the consultation, communication issues and strategies to handle stress rather than the missing content elements’.

*“I remember that the doctors were more interested in the clinical examination to know exactly what you did. They were more fastidious, in the sense: “Yes, the reflux is not all regular, you did twenty seconds instead of twenty-five seconds.” I mean, they were more like that. Whereas in the end, when I was giving feedback, it was more about the content/finally more how the exam went. Rather than on a specific point of the clinical examination (Senior student 3)*

*“I was doing a lot more feedback on how to handle their stress. About how to do communication, etc. And I think maybe that’s what’s more expected of second year students, than to really know how to do hepato-jugular reflux when they’ve never seen a patient in their life! (laughs) “(Senior student 2)*

#### A learning opportunity.

In functioning as feedback givers, the senior student s noted that by mastering the content, it stimulated their own learning and provided them with opportunities to practice the skills learned’.

*But I’m more likely to understand something I don’t understand, when I’m studying on my own. Whereas, when I know I have to be the student tutor for this formative station, I know that right now I have a student who can potentially say, “Excuse me, but what does B3 mean?” And I can’t just half understand it. So, it forces me to go a little further, to explain (Senior student 5).*

*To be able to give feedback as well. I think it’s a great way to learn to give someone feedback. You’re forced to take a step back from your own position.” (Senior student 3)*

#### Challenges as near feedback givers

The senior students also described some challenges, the main one was related on how to remain objective when giving feedback. The difficulty was to use objective criteria to assess the student’s performance beyond using a checklist.

“*Perhaps a little difficult, was in the assessments I was doing, to keep a form of objectivity*.”(Senior student 6)

Some were often afraid of saying inadequate comments while others reported that it was easier to say that they did not know and if there were elements that they were unsure of it.

“*I must admit that sometimes it can be a little stressful “because we are afraid of saying stupid things. We’re not doctors, so*...(laughs)”(Senior student 2)

They sometimes found difficult to give constructive feedback.

*“For the feedback, I sometimes have trouble finding points to improve. My feedback was too kind. “(Senior student 3) “*

## Discussion

The results from this study show that the quality of feedback given by near peers during online OSCEs was well perceived and objectively of high quality. These results are surprising given the sanitary context and stressful conditions in which these OSCEs were implemented with little time dedicated to senior students’ training as tutors.

The high scoring of the perceived quality of feedback may have been overemphasized in the pandemic context where most courses and training activities were canceled due to lack of hospital-based clinical teachers’ availability [24, 39]. Our findings are consistent with prior research which found that near peer feedback was judged to be of greater quality than input from clinically teachers, and was generally well received and accepted [40, 41]. One major strength of our study is that the near peers’ quality of feedback was assessed by analyzing the videotaped sessions and did not rely solely on perceptions.

The content addressed during the feedback slightly differed between near peers and experienced clinical teachers with senior student putting less focus on history taking and physical exam skills and elaborating less on communication/professionalism issues. The fact that near peers put less focus on physical examination can be easily explained by the fact that during this online OSCE, students were only asked to describe step by step how they would examine the patient and such format did not allow an appropriate demonstration/evaluation of physical exam skills. The reasons why they also put less emphasis on the history taking is less obvious since the duration of the feedback session was longer for near peer than for experienced clinical teachers. In addition, we do not know whether the history taking skills which were not mentioned by senior students were crucial or not to address in line with aligning with the learning objectives of the OSCE. The results from the focus groups indicate that some senior students deliberately chose to focus on different issues because of past OSCE feedback memories where the listing of physical exam elements well/poorly done or missing was experienced as fastidious. Training more specifically senior students on identifying and addressing the key skills to practice during the OSCE might be necessary beyond giving a checklist form. Finally, near peers elaborated less on communication/professionalism issues than experienced clinical teachers. This is not surprising since it requires not only clinical experience but also a frame of references that even experienced clinical teachers ignore [42, 43]. These differences in content, although statistically significant, may not be clinically relevant. It is commonly assumed that quality matters more than quantity—it may be more pedagogically relevant to address three important issues in an interactive way than five per skill domain in a directive way during a short feedback session. However, the design of our study did not allow to explore this issue. A systematic review and meta-analysis showed that students taught by peers do not have significantly different outcomes than those taught by clinical teachers when teaching relates to physical examination or communication skills [44].

Near peer feedback was experienced less stressful and more tailored to students’ needs. It represented a learning opportunity for near peers. This results are in line with the literature which shows that near peers create a less intimidating atmosphere and are more aware and realistic regarding expected knowledge and skills than clinical teachers [45]. Peer and near peer teaching is also beneficial for senior students who, by teaching, consolidate their knowledge and skills and may even improve their academic performance [22]. It also helps develop teaching skills and enhance the identity formation of future clinical teachers.

Not surprisingly, challenges reported by near peers, such as objective rating and ability to provide negative feedback are similar to those commonly described by more experienced clinical teachers [46].

### Strengths and limitations

There are several limitations to our study. First, we compared students’ perceptions and objective scores of feedbacks given by near peers and experienced clinical teachers in different formats, at different times and of different duration. These elements together with the Covid-19 pandemic context may have positively biased our results. Second, it is possible that students’ perceptions of the quality of feedback were influenced by the overall feedback including near peer, student observers and the standardized patient and not just the near peer feedback. Third, near peers represented a selection of senior students already involved in teaching activities. It is possible that we recruited only highly motivated and skilled senior students Near peer volunteered participation is indeed commonly reported in studies assessing peer/near peer assisted [20]. Involving randomly assigned senior student with no specific teaching experience may have led to lower quality feedback. Finally, the number of students as learners included in the focus groups was small and may have prevented us to capture all the perceived advantages, disadvantages and challenges of near peer feedback.

## Conclusion

A key element of feedback acceptability is the fact the source should be credible [7, 11]. This study together with other studies suggest that near peers, with limited training in teaching skills, can be considered as valuable and credible sources of feedback. Increased involvement of near peers in teaching activities is strongly supported as long as it focuses on relatively simple skills or knowledge concepts. However, training should focus both on teaching skills and the specific content of the teaching activities. Teaching of more complex skills and knowledge still requires clinical expertise and the presence of experienced tutors [47].

## Supplementary Information

**Additional file 1.**

## Data Availability

The datasets generated and/or analysed during the current study are not publicly available due to the privacy of the students but are available from the corresponding author on reasonable request.

## References

[1] Shute VJ (2008). Focus on Formative Feedback. Rev Educ Res.

[2] Crommelinck M, Anseel F (2013). Understanding and encouraging feedback-seeking behaviour: a literature review. Med Educ.

[3] Casey PM, Goepfert AR, Espey EL, Hammoud MM, Kaczmarczyk JM, Katz NT, Neutens JJ, Nuthalapaty FS, Peskin E, G. Association of Professors of, C. Obstetrics Undergraduate Medical Education (2009). To the point: reviews in medical education–the Objective Structured Clinical Examination. Am J Obstet Gynecol.

[4] Miller A, Archer J (2010). Impact of workplace based assessment on doctors’ education and performance: a systematic review. BMJ.

[5] Norcini J, Burch V (2007). Workplace-based assessment as an educational tool: AMEE Guide No. 31. Med Teach.

[6] Schuwirth L, van der Vleuten C, Durning SJ (2017). What programmatic assessment in medical education can learn from healthcare. Perspect Med Educ.

[7] Archer JC (2010). State of the science in health professional education: effective feedback. Med Educ.

[8] Watling C (2014). Resident teachers and feedback: time to raise the bar. J Grad Med Educ.

[9] Wiese A, Kilty C, Bennett D (2018). Supervised workplace learning in postgraduate training: a realist synthesis. Med Educ.

[10] Weallans J, et al. Postgrad Med J 2022;98:138–149. doi:10.1136/postgradmedj-2020-139566. 10.1136/postgradmedj-2020-139566 33563716

[11] Eva KW, Armson H, Holmboe E, Lockyer J, Loney E, Mann K, Sargeant J (2012). Factors influencing responsiveness to feedback: on the interplay between fear, confidence, and reasoning processes. Adv Health Sci Educ Theory Pract.

[12] Eaton D, Sargeant S (2012). Maturational differences in undergraduate medical students’ perceptions about feedback. Med Educ.

[13] Van de Ridder JM, Berk FC, Stokking KM, ten Cate OT (2015). Feedback providers’ credibility impacts students’ satisfaction with feedback and delayed performance. Med Teach.

[14] Chang YC, Lee CH, Chen CK, Liao CH, Ng CJ, Chen JC, Chaou CH (2017). Exploring the influence of gender, seniority and specialty on paper and computer-based feedback provision during mini-CEX assessments in a busy emergency department. Adv Health Sci Educ Theory Pract.

[15] Fernando N, Cleland J, McKenzie H, Cassar K (2008). Identifying the factors that determine feedback given to undergraduate medical students following formative mini-CEX assessments. Med Educ.

[16] JunodPerron N, Louis-Simonet M, Cerutti B, Pfarrwaller E, Sommer J, Nendaz M (2016). The quality of feedback during formative OSCEs depends on the tutors’ profile. BMC Med Educ.

[17] Hunter AJ, Desai SS, Harrison RA, Chan BK (2004). Medical student evaluation of the quality of hospitalist and nonhospitalist teaching faculty on inpatient medicine rotations. Acad Med.

[18] Kripalani S, Pope AC, Rask K, Hunt K, Dressler DD, Branch WT, Zhang R, Williams MV (2004). Hospitalists as teachers. J Gen Intern Med.

[19] Bulte C, Betts A, Garner K, Durning S (2007). Student teaching: views of student near-peer teachers and learners. Med Teach.

[20] Friel O, Kell D, Higgins M (2018). The evidence base for peer assisted learning in undergraduate medical education: a scoping study. MedEdPublish.

[21] Atwater MM (1996). Social constructivism: infusion into the multicultural science education research agenda. J Res Sci Teach.

[22] Williams B, Reddy P (2016). Does peer-assisted learning improve academic performance? A scoping review. Nurse education today.

[23] Lockspeiser TM, O’Sullivan P, Teherani A, Muller J (2008). Understanding the experience of being taught by peers: the value of social and cognitive congruence. Adv Health Sci Educ Theory Pract.

[24] Khan R, Payne MWC, Chahine S (2017). Peer assessment in the objective structured clinical examination: A scoping review. Med Teach.

[25] Hannan TA, Umar SY, Rob Z, Choudhury RR (2021). Designing and running an online Objective Structured Clinical Examination (OSCE) on Zoom: a peer-led example. Med Teach.

[26] Gulati RR, McCaffrey D, Bailie J, Warnock E. Virtually prepared! Student-led online clinical assessment. Educ Prim Care 2021:1–2. 10.1080/14739879.2021.1908173 33843480

[27] Roberts V, Malone K, Moore P, Russel-Webster T, Caufield R (2020). Peer teaching medical students during a pandemic. Med Educ Online.

[28] Rosenthal HB, Sikka N, Lieber AC, Sanky C, Cayon C, Newman D, Marquez DR, Ziff J, Blum JR, Dai JB, Groden P, Pasik S, Pour T (2020). A Near-Peer Educational Model for Online, Interactive Learning in Emergency Medicine. West J Emerg Med.

[29] Zoom; https://zoom.us. Accessed 20 June 2021.

[30] Junod Perron N, Nendaz M, Louis-Simonet M, Sommer J, Gut A, Baroffio A, Dolmans D, van der Vleuten C (2013). Effectiveness of a training program in supervisors’ ability to provide feedback on residents’ communication skills. Adv Health Sci Educ Theory Pract.

[31] Hattie J, Timperley H (2007). The power of feedback. Rev Educ Res.

[32] Kurtz S, Silverman J, Draper J (2005). Teaching and learning communication skills in medicine.

[33] Cantillon P, Sargeant J (2008). Giving feedback in clinical settings. BMJ.

[34] Van Thiel J, Kraan HF, Van Der Vleuten CP (1991). Reliability and feasibility of measuring medical interviewing skills: The revised Maastricht history-taking and advice checklist. Med Educ.

[35] Krueger RA, Casey MA (2000). Focus groups: A practical guide for applied research.

[36] Braun V, Clarke V (2006). Using thematic analysis in psychology. Qual Res Psychol.

[37] Kiger ME, Varpio L (2020). Thematic analysis of qualitative data: AMEE Guide No. 131. Med Teach.

[38] M.A.t.s. Dowling, Atlas.ti (software), in: L.M. Given (Ed.), The SAGE encyclopedia of qualitative research methods Sage Publications 2008, p. 37.

[39] Schwill S, Fahrbach-Veeser J, Moeltner A, Eicher C, Kurczyk S, Pfisterer D, Szecsenyi J, Loukanova S (2020). Peers as OSCE assessors for junior medical students - a review of routine use: a mixed methods study. BMC Med Educ.

[40] Moineau G, Power B, Pion AM, Wood TJ, Humphrey-Murto S (2011). Comparison of student examiner to faculty examiner scoring and feedback in an OSCE. Med Educ.

[41] Reiter HI, Rosenfeld J, Nandagopal K, Eva KW (2004). Do clinical clerks provide candidates with adequate formative assessment during Objective Structured Clinical Examinations?. Adv Health Sci Educ Theory Pract.

[42] Kogan JR, Conforti LN, Iobst WF, Holmboe ES (2014). Reconceptualizing variable rater assessments as both an educational and clinical care problem. Acad Med.

[43] Levinson W (2011). Patient-centred communication: a sophisticated procedure. BMJ Qual Saf.

[44] Rees EL, Quinn PJ, Davies B, Fotheringham V (2016). How does peer teaching compare to faculty teaching? A systematic review and meta-analysis. Med Teach.

[45] de Menezes S, Premnath D (2016). Near-peer education: a novel teaching program. Int J Med Educ.

[46] Kogan JR, Conforti LN, Bernabeo EC, Durning SJ, Hauer KE, Holmboe ES (2012). Faculty staff perceptions of feedback to residents after direct observation of clinical skills. Med Educ.

[47] Knobe M, Holschen M, Mooij SC, Sellei RM, Munker R, Antony P, Pfeifer R, Drescher W, Pape HC (2012). Knowledge transfer of spinal manipulation skills by student-teachers: a randomised controlled trial. Eur Spine J.

