# Supplementary material for: The pedagogical value of near-peer feedback in online OSCEs
Source: BMC Med Educ. 2022 Jul 25;22:572. doi: 10.1186/s12909-022-03629-8 (PMC9310367; doi:10.1186/s12909-022-03629-8)
Supplement: Supplementary file 1 — Additional file 1. [file 12909_2022_3629_MOESM1_ESM.docx]

The pedagogical value of near-peer feedback in online OSCEs

Sader Julia^1^, Cerutti Bernard^1^, Meynard Louise^2^, Geoffroy Frédéric^1^, Meister Véronique^2^. Paignon Adeline^2,3^ & Junod-Perron Noëlle^1^

# Appendix A focus group guide

# Online focus groups interview guide Student learners and student tutors

Aim:

The purpose of this study is to evaluate the value of online formative stations with simulated patients, the contribution of feedback given to students by student monitors, and the value of incorporating observers and feedback givers. The study is part of the quality assessment loop for innovative teaching methods.

- Social phase
- Introduction of moderators and their roles
- Description of the purpose of the project
- Introduction of participants
- Rules on group functioning

**First question**: "Can you reflect on your experience at the online formative station and define for yourself what was most helpful to your learning. ".

- Receiving online feedback how did you experience it?

- Share your key words

- Try to give examples that illustrate your point

**Second question**: "Okay, thank you, so now I'm going to ask you to reflect on your experience of being observed by a student instructor and define for yourself what was most helpful to your learning. ".

- Feedback from one of your peers - how did it go?
- Share your key words
- Try to give examples that illustrate your point

**Third question**: "Very good, and now can you reflect on your experience as an observer at this formative station and was it useful for your learning? "[JS1]

- Feedback from the simulated patient how did you perceive it?
- Share your key words
- Try to give examples that illustrate your point

**Fourth question:** "What was the value of this format for you? Do you have any suggestions or ideas for improving this format? "

**Fifth question:** Did you feel confident in the online sessions?

- distance
- peer evaluation
- logistical aspects of videoconferencing

**Sixth question:** 11. Did the interaction with the patient seem credible to you?

- personal involvement
- getting into the game
- play of the SP
- - vision focused on the person's face and little on the rest of the body

**Seventh question:** 12. "Did you encounter any difficulties during this formative station? If so, can you describe them for us?

- Do you have any comments about having to describe the physical examination you would do to the SP?
  - usefulness in helping to remember the gestures
  - frustration of not being able to do, not being able to touch
- Summarize
- Check if there are other elements to discuss
- Close by thanking them and informing them that they will receive a summary of the discussion in a few weeks for comment
